# The development and evolution of the Irish Hip Fracture Database: a quality care initiative 2013–2024

**DOI:** 10.1007/s11657-026-01731-8

**Published:** 2026-06-25

**Authors:** L. Brent, E. Ahern, T. Murphy, C. Hurson, T. Coughlan, P. Hickey, C. Lodola, M. E. Walsh, E. Byrne, F. Fitzpatrick

**Affiliations:** 1 School of Population Health, Royal College of Surgeons in Ireland, University of Medicine and Health Sciences, Dublin, Ireland; 2 National Office of Clinical Audit, Royal College of Surgeons in Ireland, Dublin, Ireland; 3 Department of Geriatric Medicine, Cork University Hospital, Cork, Ireland; 4 Department of Orthopaedic Surgery, University Hospital Waterford, Waterford, Ireland; 5 Department of Orthopaedic Surgery, St. Vincent’s University Hospital, Dublin, Ireland; 6 Discipline of Medical Gerontology, School of Medicine, Trinity College Dublin, Dublin, Ireland; 7 Department of Age-Related Healthcare, Tallaght University Hospital, Dublin, Ireland; 8 Centre for Positive Health Sciences, Royal College of Surgeons in Ireland, University of Medicine and Health Sciences, Dublin, Ireland; 9 Department of Clinical Microbiology, Royal College of Surgeons in Ireland, University of Medicine and Health Sciences, Dublin, Ireland; 10 Department of Clinical Microbiology, Beaumont Hospital, Dublin, Ireland

**Keywords:** Hip fracture clinical audit, Governance, Quality improvement

## Abstract

***Summary*:**

Clinical audits are central to quality improvement in healthcare, yet practical real-world descriptions of how they are implemented, governed and sustained are limited. This paper describes the establishment, evolution and impact of the Irish Hip Fracture Database (IHFD) within the Irish healthcare system as an exemplar.

**Introduction:**

Clinical audits compare clinical practice against defined standards to identify areas for improvement and showcase excellence. International literature provides limited insight into how they are governed and sustained. Using the IHFD, a mature national audit, this paper reviews its development, evolution and sustained momentum from 2013 to 2024.

**Methods:**

Longitudinal data were captured via the Hospital Inpatient Enquiry (HIPE) system, encompassing all eligible Irish acute hospitals. Included cases were patients aged ≥ 60 years with hip fractures, as defined by IHFD. Three organisational surveys (2016, 2020, 2024) were undertaken to assess hospital resources, care pathways, governance structures and audit supports.

**Results:**

In total, 41,304 cases were included in the analysis. National coverage exceeded 90% from 2017. Demographic characteristics remain consistent. Improvements in data quality, adherence to Irish Hip Fracture Standards were observed, including geriatrician assessment (11% in 2013 to 86% in 2024) and bone health assessment (65% in 2013 to 90% in 2024). Service reconfigurations and resourcing to support hip fracture care occurred over this period. Engagement with the audit remained robust despite major challenges including COVID-19, a national cyberattack and ongoing health system reform.

**Conclusion:**

Sustained success of a national hip fracture clinical audit requires strong leadership, effective governance, clear clinical standards, accessible data and timely reporting. Continued relevance requires agility and alignment with evolving system needs.

**Supplementary Information:**

The online version contains supplementary material available at 10.1007/s11657-026-01731-8.

## Introduction

Clinical audits systematically compare clinical practice against defined standards and are a cornerstone of quality improvement (QI) in healthcare services. In particular they identify areas for improvement and showcase excellence. Healthcare systems thrive when there is a culture of QI embedded [1]. International literature however, provides limited insight into how clinical audits are governed and sustained.

The Irish Hip Fracture Database (IHFD) was established in 2012 under the governance of the National Office of Clinical Audit (NOCA) [2]. The goal was to improve clinical quality and organisational effectiveness in hip fracture care, benchmark outcomes against international standards and support continuous improvement. Delivering efficient, high-quality care can reduce costs and reduce reccurrence rates. Evidence from the UK National Hip Fracture Database (NHFD) demonstrated that national clinical audits can drive improvements in care and reduce mortality [3]. The governance of clinical audits is essential to provide accountability and ensure there is QI embedded in healthcare in a systematic way [4]. Using a published governance model can strengthen reporting.

International comparisons across hip fracture audits can provide insights for standardising care and promoting QI [5]. Globally, seventeen national hip fracture clinical audits/registries exist, with IHFD considered a mature example. In 2021, ten audits developed a minimum common dataset (MCD) supported by the Fragility Fracture Network (FFN) [6]. This was expanded in 2023 to assess compliance with the MCD [7]. Despite similar standards, variations exist, limiting direct comparability and federated analysis for research and service improvement. At the 2025 Global FFN Congress, representatives from seventeen national audits gathered, the largest meeting of its kind, to discuss challenges in audit establishment, funding models, maintaining momentum and relevance [8].

This paper describes the establishment and evolution of the IHFD using a longitudinal, descriptive approach structured by the SQUIRE 2.0 Guidelines, which support transparent and comprehensive reporting of quality-improvement initiatives [9]. As a national clinical audit that has matured within the Irish health system, the IHFD provides a practical exemplar for understanding how clinical audits are developed, governed, and sustained. Building on a previous international scoping review of hip fracture audit governance and reports of Irish trends in hip fracture care [10], this paper outlines a detailed account of how a national hip fracture audit can be established and maintained. Using the core governance elements for national hip fracture clinical audits [11] (Appendix 1), we describe the IHFDs structure, implementation process and outcomes, providing a practical guide for similar national clinical audit initiatives.

Specific aims the following:

1. To describe the governance and operational structure of an established national clinical audit using predefined governance elements
2. To report descriptive longitudinal trends in patient demographics, service provision, and clinical standards from 2013 to 2024
3. To demonstrate how audit governance can support sustained QI over time

## Methods

This paper describes the establishment and evolution of the IHFD using a longitudinal, descriptive approach structured by the SQuIRE V2.0 Guidelines [9].

### Context

National Clinical Audits (NCA) evaluate clinical practice against defined standards and are useful for identifying areas for improvement and examples of excellence [1]. In Ireland, national clinical audits are commissioned by the Health Service Executive (HSE), National Centre of Clinical Audit (NCCA) via the Chief Clinical Officer. NOCA, established in 2012, now oversees the largest portfolio of NCA’s in the Irish Health System. Based at the Royal College of Surgeons in Ireland (RCSI), it operates under an independent voluntary board.

### About the Irish Hip fracture database

The proposal for the IHFD originated in 2009 through collaboration between leaders in Geriatric Medicine and Orthopaedics, representing the Irish Gerontological Society (IGS) and the Irish Institute of Trauma and Orthopaedic Surgery (IITOS). The embryonic IHFD was developed on an industry-funded database before moving into NOCA in 2012.

**Fig. 1:**
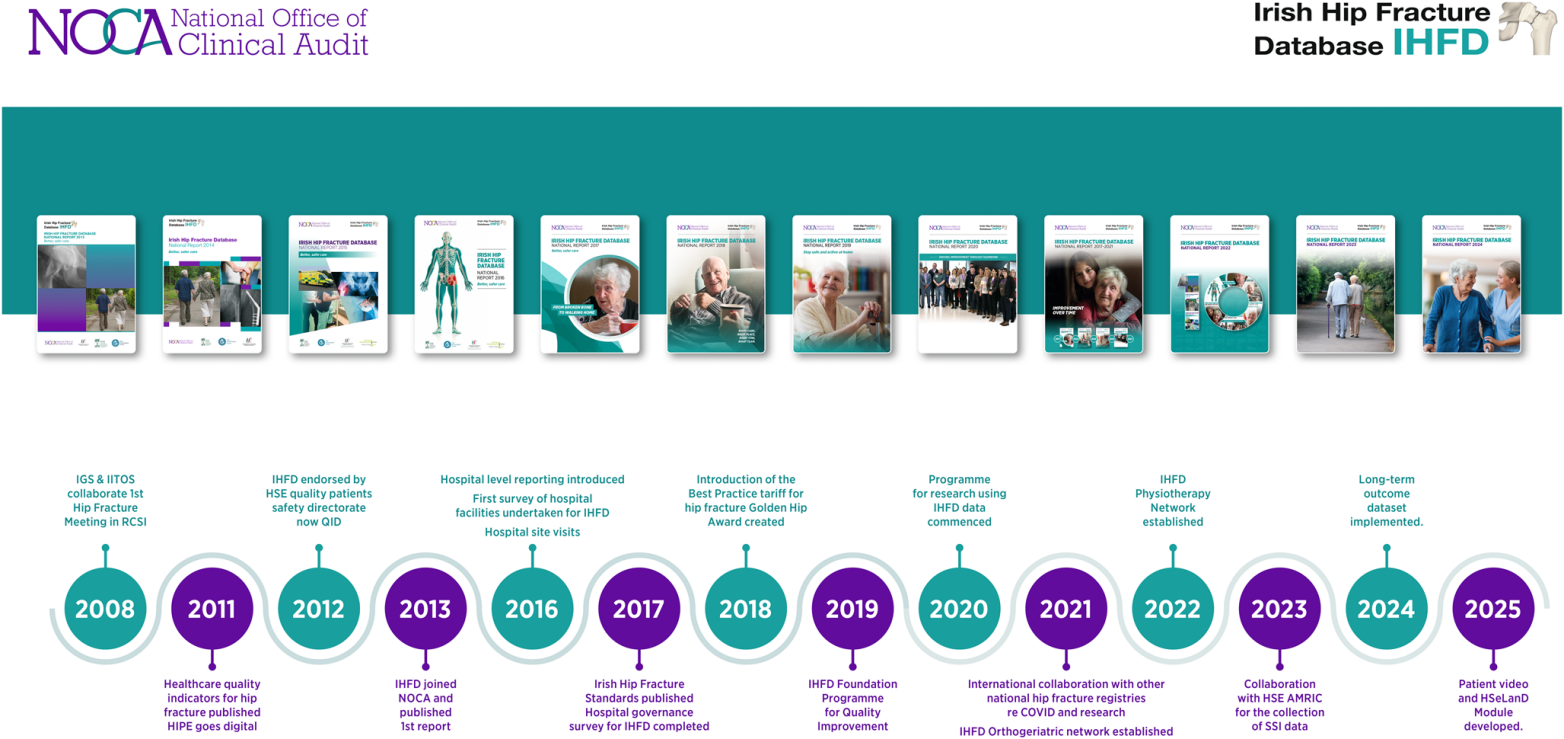
Timeline and evolution of the IHFD

The IHFD is now a mature national audit with twelve years of reporting, including > 43,000 cases and consistently high data quality completeness with > 90% captured annually. All 16 eligible hospitals participate, each supported by a clinical lead, audit coordinator and a hospital hip fracture governance committee. In addition, three organisational surveys were conducted to describe the resources, care pathways, governance structures and audit support’s within participating hospitals. Figure 1 shows the IHFDs evolution since inception.

### Intervention

The measures in this paper follow a descriptive, observational design, using longitudinal patient-level data from 2013 to 2024 and repeated organisational audits.

Measures include the following: Patient-level measures (demographics, clinical standards adherence, outcomes, data quality); organisational-level measures (service availability, staffing/models of care and infrastructure changes).

## Content of audit

**Fig. 2:**
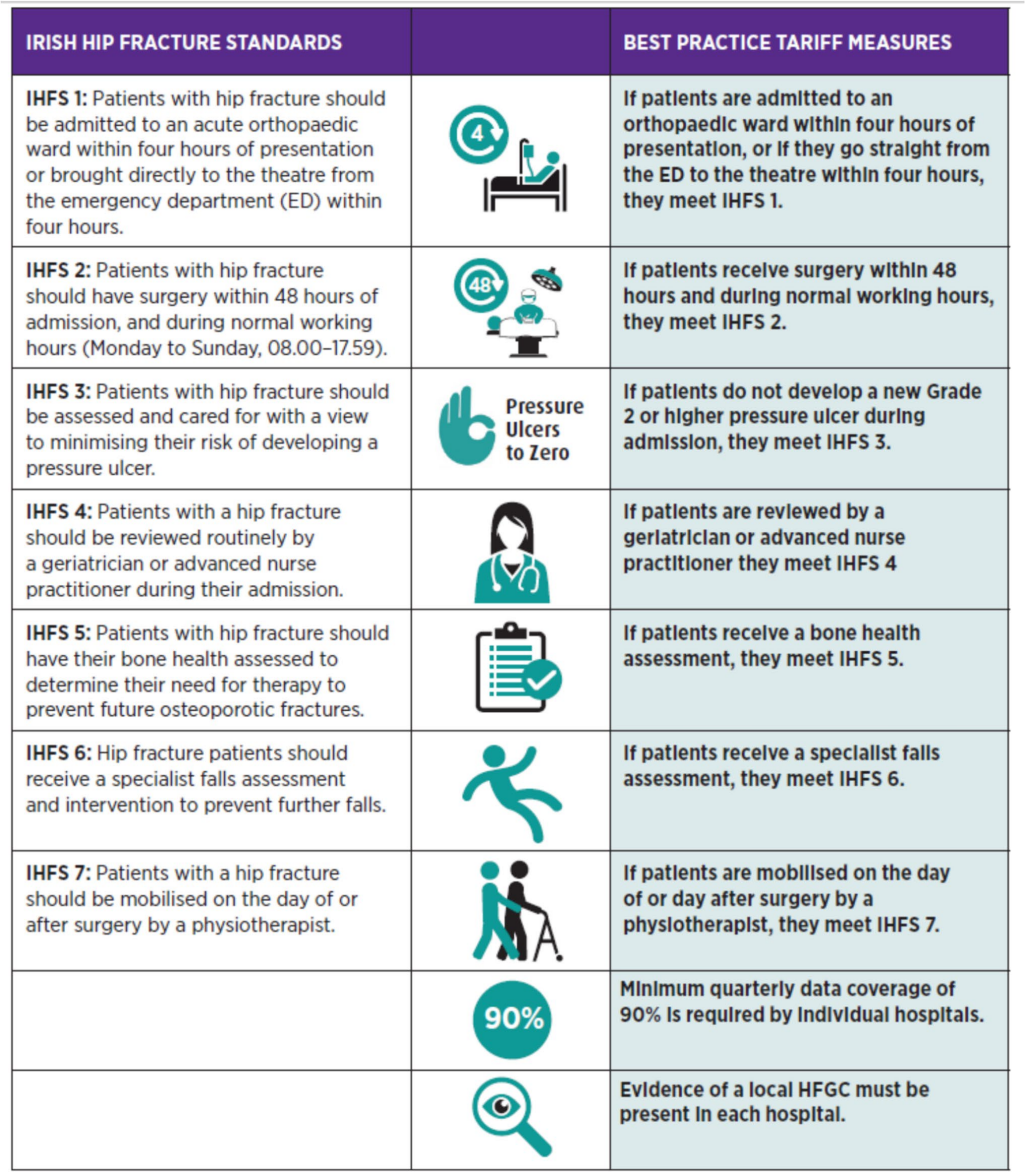
Irish Hip Fracture Standards (IHFS) and best practice tariff measures

The initial IHFD dataset was based on the UK NHFD [12] and its six “Blue Book Standards” from the British Orthopaedic Association and British Geriatric Society “Blue Book” guidelines [13]. As these evidence-based standards evolved, the IHFD published the Irish Hip Fracture Standards (IHFS) in 2017 comprising seven core clinical measures [14] (Fig. 2).

The dataset has continued to evolve to capture emergent variables relevant to hip fracture care such as delirium screening, nutritional screening and nerve block administration [15]. Amendments occur at year-end to ensure complete annual data capture.

In 2018, Ireland introduced its first best practice tariff (BPT) for hip fracture care, a €1000 payment per case meeting the IHFS and two data quality requirements, namely having a hip fracture governance committee and submission of 90% of data for that reporting period (Fig. 2) (the 90% target indicates that data has been entered on 90% of eligible cases; however it does not indicate that high compliance has been achieved in the individual clinical standards). This financial incentive aimed to encourage best practice and reward hospitals achieving both clinical and data quality standards. A framework for local hospital governance was provided to each hospital (Appendix 1).

### Inclusion criteria for IHFD

Cases identified in the Hospital Inpatient Enquiry (HIPE) system, with a hip fracture coded under ICD-10-AM diagnosis codes S72.00 to S72.2 or a specified fracture type (e.g. intracapsular displaced/undisplaced, intertrochanteric or subtrochanteric) excluding periprosthetic fractures.

Cases aged ≥ 60 years.

## Management

### Establishing the IHFD

Between 2012 and 2013, the IHFD was managed by an orthopaedic nurse specialist and a geriatrician both balancing full-time clinical roles. Following the establishment of NOCA in 2013, operational support became available. In 2015 a dedicated audit manager (0.4 WTE), an orthopaedic nurse specialist was appointed to lead IHFD operations. Core responsibilities included the following: Creating an IHFD handbook with data and clinical definitions, user guidance, creating resources to build skills in data analysis, reporting and QI. In 2016, the IHFD audit manager and clinical lead conducted hospital visits to review hip fracture pathways, identify local innovations and troubleshoot service challenges [16]. These visits strengthened engagement and informed governance priorities. Monthly virtual meetings with hospital audit coordinators addressed data collection, quality, and pathway issues, complemented by annual workshops and targeted masterclasses on relevant topics which became routine components of audit support.

### NOCA IHFD governance committee

Under NOCA governance, the IHFD governance committee was established. This committee is clinically led with joint leadership from orthopaedics and geriatrics, supported by representatives from emergency medicine, nursing, health and social care, research, public health, hospital management, audit coordinators, national clinical programmes and societies and two patient and public interest (PPI) representatives. An executive NOCA representative and administrator ensure operational continuity.

The committee have an agreed terms of reference and meet quarterly to review outputs, data quality, hospital participation, outliers, reporting, dataset/standards amendments and research proposals. The audit governance structure relies on strong leadership and guidance. The clinical leads, represent their national societies and provide unfunded, yet highly engaged leadership. This proactive approach has been critical to sustaining engagement and driving service improvements.

Central NOCA supports include access to an analytical team, administration, information governance and IT, communication and events facilitating national reporting and dissemination through virtual or in-person events, social media and press releases. Participation in the IHFD is voluntary but strongly incentivised by the BPT. The annual *Golden Hip Award* recognises the hospital with the highest BPT compliance and has become a highly valued incentive, that further strengthens engagement.

## Implementation process

Figure 1 illustrates the IHFD evolution. Initial implementation followed a stakeholder town hall at the RCSI, attended by orthopaedic surgeons, geriatricians, nurses, health and social care professionals with early adoption driven by local champions. Transitioning the IHFD into NOCA strengthened governance and accelerated hospital participation.

Sixteen hospitals were identified as eligible for inclusion, each required to appoint a clinical lead and audit coordinator. Engagement was further supported by the introduction of a national key performance indicator (KPI) for hip fracture care [17]. While initial participation was limited by resource constraints, 12 hospitals contributed to the first report, coverage expanded to all 16 hospitals by 2015 Appendix 3.

By 2016, enhanced resources within NOCA including a dedicated 0.5 WTE audit manager enabled hospital-level reporting, reflecting improved data completeness and quality [16]. The IHFD manager provided peer support and training, delivered workshops, and developed resources to support data entry and quality including a handbook with a data dictionary, definitions and reporting guidance.

Annual hip fracture clinical conferences were established and in 2017, the IHFD successfully bid to host the global Fragility Fracture Network Conference in the RCSI. Over 500 international delegates attended this event in 2018 [9]. That year was pivotal for the IHFD, with the introduction of the BPT and the Golden Hip Award, recognising hospitals with the highest compliance with clinical standards.

Throughout the COVID-19 pandemic (2020–2022), strong virtual engagement with hospitals was maintained. In 2023, publication of the tenth national report coincided with the resumption of in-person events at HIPFEST celebrating a decade of data and featuring international speakers including the founders of the UK NHFD.

Subsequently, two networks (orthogeriatric and physiotherapy) were established to strengthen focus on key areas of hip fracture care and foster multidisciplinary collaboration. Meeting regularly both virtually and in-person, these networks adopted a co-design approach to develop consensus, resources and prioritise care strategies, generating significant collaborations and resources for healthcare staff and patients [18].

## Technical components

The IHFD is incorporated within the HIPE system, the principal source of information for all publicly funded acute hospitals in Ireland, managed by the Healthcare Pricing Office (HPO) [19]. HIPE data merges with IHFD data, including demographics and ICD-10-AM codes reducing data entry burden and enhancing validation. Audit coordinators and clinical leads have secure access to the IHFD portal, facilitating real-time reporting and engagement at hospital level.

The HPO provides quarterly data extracts to NOCA, which are used to update dashboards. Hospitals receive quarterly data validation reports and local teams can export data in Comma-Separated Values (CSV) format for further analysis at any time.

## Data entry process

At each participating hospital, designated audit co-coordinators (usually orthopaedic nurses) enter data into the IHFD portal retrospectively. Eligible hip fracture cases are identified using discharge reports generated from HIPE, supplemented by manual review in accordance with inclusion/exclusion criteria. Data entry usually occurs after patient discharge and includes variables describing the patient pathway, pre-fracture health status, the IHFS and outcomes such as length of stay, discharge destination, and follow up at 30, 120 and 365 days. The current dataset is available in the latest NOCA report [15].

## Data quality

IHFD data quality is assessed within NOCA using internationally agreed dimensions defined by the Health Quality Information Authority [20]. Support measures include a data dictionary, help-text and explanations within the HIPE portal, monthly teleconferences between the audit manager and hospital coordinators, annual workshops and supplementary educational materials. Quarterly data validation reports (DVRs) accompany IHFD reports to facilitate local validation. Each annual report includes a formal statement on data quality.

## Reporting/data dissemination

Dissemination of IHFD data has evolved, with intentional strategies to engage hospitals. Between 2013 and 2016, several reports were integrated into the portal to support case identification including:

- Discharge report identifying cases that have been coded as a hip fracture
- Data completeness report
- Summary report of key data
- Hip Fracture Standards report
- Data export

An annual national report summarising national and hospital-level data is launched at the yearly hip fracture conference, attended by key stakeholders. Public-facing materials such as infographics, vector graphics (e.g. Fig. 2) and summary reports were introduced to enhance accessibility and wider engagement.

From 2017 onwards, infographics became synonymous with IHFD reporting, contributing to increased audit visibility. In 2019, Microsoft Power BI (business intelligence) dashboards were introduced enabling hospital-level data visualisation to support quality assurance and improvement. Reporting formats were co-designed with stakeholders, including PPI representatives to ensure relevance. Quarterly dashboards provide hospitals with data on quality, compliance with IHFS and BPT performance with iterative refinements based on stakeholder feedback. These dashboards had a positive impact, serving as a foundation for discussion at local HFGC meetings. Compliance trends for each standard are displayed and analysed using statistical process control charts (SPC) to guide improvement [21].

### Quality improvement

In 2019, a foundation QI programme was embedded within the IHFD in collaboration with the HSE Quality and Patient Safety Directorate, [21]. Dashboard-style reports were aligned with QI methodologies [22], enabling hospital staff to conduct QI projects more effectively. By 2023, all 16 hospitals had direct access to live dashboards, supporting real-time, data-driven decision-making. All public-facing reports, including summary reports are co-designed with PPI representatives to ensure relevance and clarity.

## Accessibility of data

Hospitals have full access to raw IHFD data, which can be exported into Excel for local analysis. NOCA operates a formal data-access procedure for secondary use of audit data, requiring ethical approval for research. Other options include data access for QI, service evaluation, health system reconfiguration and resource planning [23]. To date, IHFD data have supported over thirty peer-reviewed publications, including Health Research Board-funded projects such as the HipFORGE (Hip Fracture Outcome Recording and Geographic Equality), HipFORESST (Hip Fracture Outcome Recording: Empowering, Supporting and Sustaining Teams) and HipCog (evaluating the impact of cognitive impairment on outcomes for older adults with hip fracture) [24–28].

### Analysis

A descriptive analysis of IHFD data from 1 st January 2013 to 31 st December 2024 is presented focusing on data quality, patient demographics, compliance with hip fracture standards and outcomes. The objective is to evaluate the audits’ impact on clinical outcomes, system-level change, QI activity and policy development.

### Ethical considerations

Ethical approval was granted for analysis of audit data and organisational surveys by RCSI [REC 202405014]. The lead author’s (LB’s) longstanding role in establishing and managing the IHFD since its inception offers unique contextual insight into operational processes underpinning audit sustainability and impact. Drawing on a background in orthopaedic nursing, alongside national and international roles with the International Collaboration of Orthopaedic Nursing and the Global Fragility Fracture Network, supports the depth of understanding, facilitates shared learning and strengthens relevance of findings for other audits.

## Results

This analysis includes data on 41,304 hip fracture cases recorded in the IHFD between 1 st January 2013 and 31 st December 2024.

### Data quality

Table 1 illustrates the progressive increase in case ascertainment from 1950 cases in 2013 (representing 63% national coverage) to 4293 cases in 2024 (representing 95% national coverage). By 2015, all hospitals were participating. Data quality has remained > 90% since 2017, coinciding with the announcement of the forthcoming 2018 BPT, which introduced a data quality standard of 90%. This data quality standard was maintained despite the impact of the global COVID-19 pandemic and a subsequent national cyberattack on the Irish Health system in 2021 [29]. It is indicative of good engagement with the audit.

**Table 1:** Data coverage and quality, Irish Hip Fracture Database, 2013–2024

Data quality			
Year	**% of participating hospitals**	**Number included in audit**	**National coverage**
2013	75%	1950	63%
2014	88%	2664	78%
2015	100%	2962	82%
2016	100%	3159	88%
2017	100%	3497	97%
2018	100%	3751	99%
2019	100%	3701	99%
2020	100%	3666	99%
2021	100%	3806	99%
2022	100%	3909	94%
2023	100%	3983	93%
2024	100%	4293	95%

### Patient characteristics

The demographic profile of hip fracture patients has remained relatively stable of the duration of the IHFD. (Appendix 3) Two-thirds of hip fracture cases are female (67%), and 58% are aged over 80 years. The majority are admitted from home (84%) although there has been a gradual increase in admissions from nursing homes (7% in 2013 to 11% in 2024). One of the most significant changes in the patient profile is the proportion of patients recorded with an ASA score of 3 or above indicating severe systemic disease (42% in 2013 to 65% in in 2024).

### Irish hip fracture standards

**Fig. 3:**
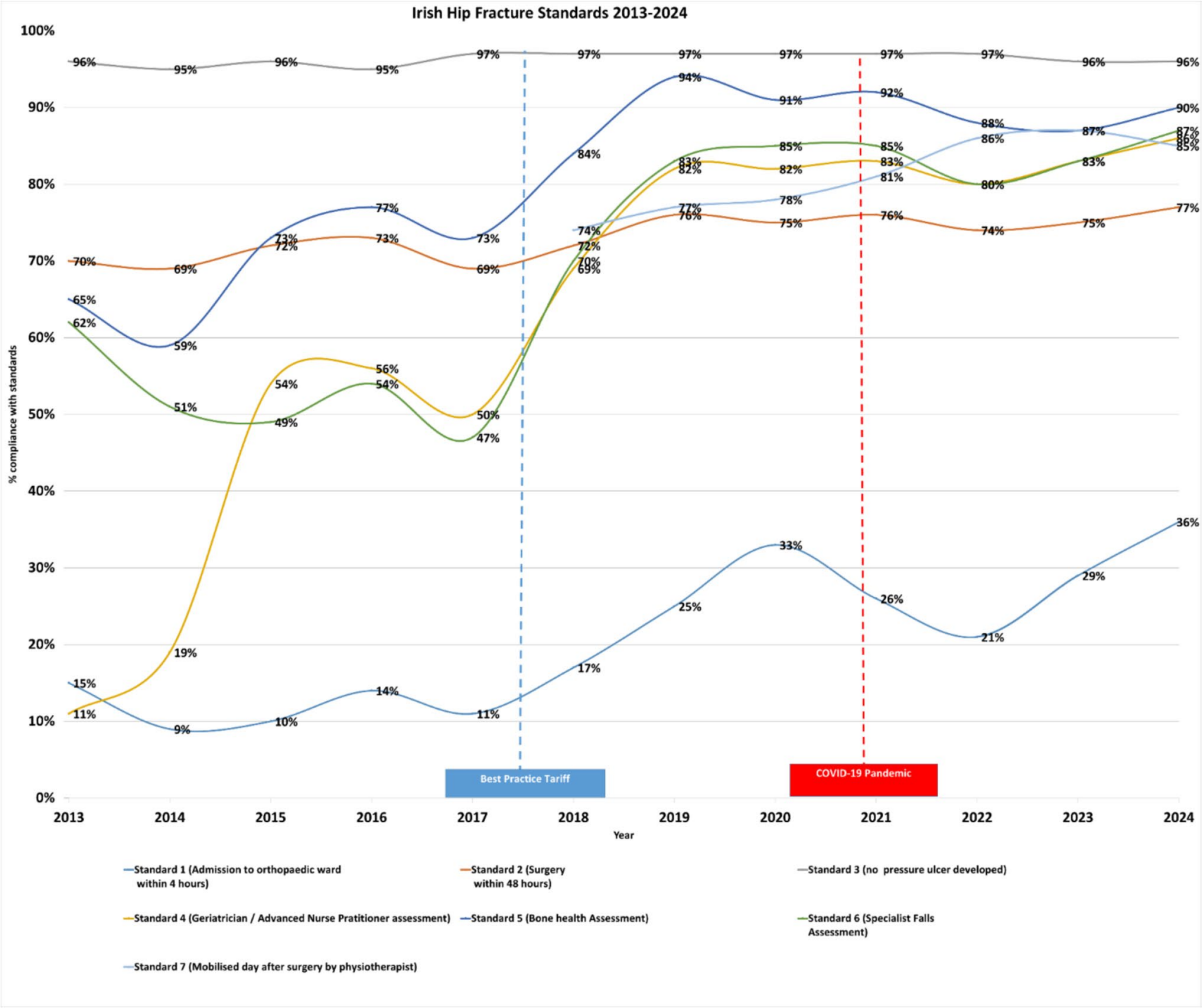
Compliance with Irish Hip fracture Standards 2013–2024

Compliance with the IHFS standards has improved significantly between 2013 and 2024 (Fig. 3). Admission from an emergency department to an orthopaedic ward increased from 15% in 2013 to 36% in 2024. The proportion of patients receiving surgery within 48 h increased from 70 to 77%. Rates of pressure ulcer prevention remained high at 96% Improvements were also observed in geriatrician assessment (increased from 11 to 86%), bone health assessment (increased from 65 to 90%), and falls assessment (increased from 62 to 87%). Since the introduction of the day one physiotherapy mobilisation standard in 2018, compliance increased from 74 to 85%.

There was a clear pattern of improvement aligned with the establishment of the IHFD between 2013 and 2017; however the trajectory of improvement accelerated significantly following the signalling of the BPT in 2017 (Fig. 3). As a consequence, the majority of hip fracture standards have been maintained at a very high level. This period also coincided with the creation of orthogeriatric consultant posts, a speciality previously absent in Ireland. Once the BPT was signalled to the health service, many orthogeriatric posts were approved as hospitals recognised that they could not meet the BPT standards without that expertise.

Compliance with the BPT has increased three-fold from 7% in 2018 to 21% in 2024. To date, almost €4 million has been distributed to hospitals for reinvestment in trauma services, as determined by local hip fracture governance committees (Appendix 2).

Overall, the combination of defined standards, transparent reporting and a financial incentive has aligned with the observed improvements.

### Outcomes

The IHFD reports on several outcome metrics, all of which have shown positive trends. Discharges to rehabilitation services increased from 29% in 2018 to 36% in 2024. The proportion of patients discharged directly home increased during the same period from 20 to 22%. Overall the increase in patients being discharged home directly has not been significant; however more patients are being discharged to an inpatient rehabilitation service which is a positive step and a direct result of having orthogeriatric services established to coordinate this discharge pathway properly. Also, patients who were formally admitted from nursing homes and discharged directly back there, are now being offered a rehabilitation pathway before discharge back to nursing home. Currently, in Ireland, early supported discharge teams are only being established so it is expected more patients will have the opportunity to be discharged directly home with the community supports available to see them in their own home.

The percentage of patients requiring new admission to long-term care halved, decreasing from 6 to 3%. Other discharge destinations include but are not limited to return admissions to long-term care, convalescence and palliative care/hospice care. Clinical outcomes also improved. Inpatient mortality fell from 5 to 4%, while both mean and median lengths of hospital stay reduced from 20 to 19 days (mean) and 13 to 12 days (median) respectively. This is not an insignificant reduction when you consider a saving of one day’s length of stay with the average cost of a hospital bed day being approximately €1,195. As an example in 2024, 4,294 cases were recorded in the national report so this would be a saving of over €5 million with a 1-day reduction [30].

### Local hospital governance and resources

Organisational audits conducted in 2016, 2020, and 2024 demonstrate improvements in hip fracture care pathways including the national introduction of the hip fracture bypass protocol, enabling ambulance services to transport patients directly to the operating hospital [15]. Compliance with this pathway increased from 87% in 2013 to 96% in 2024.

Since 2020, all hospitals have established hip fracture governance committees in line with IHFD standards. The number of trauma beds has increased nationally from 477 in 2016 to 550 in 2024. In addition, three quarters (n = 12) of trauma services maintain a protected hip fracture bed to receive incoming patients promptly. Orthogeriatric services have expanded more than seven-fold, rising from 33.5 h per week in 2016 to over 247 h in 2024. An even greater increase was observed in hip fracture care advanced nurse practitioner posts from none in 2016 to 20 in 2024. Weekend provision of physiotherapy services has increased from 7 to 12 services, representing more than a 1.5-fold increase in weekend cover. Since the rollout of the QI foundation course, participating hospitals have increasingly adopted a culture of QI using the IHFD. A particular highlight for the IHFD in 2023 was the showcase of 44 QI projects at the HIPFEST conference in 2023, demonstrating the scale and maturity of QI engagement [31, 32].

### Next steps

The data presented above are a high-level descriptive overview of the data quality, patient characteristics, adherence to the hip fracture standards, outcomes and changes in resources. As a consequence of a number of the clinical standards (4–6, i.e. percentage seen by a geriatrician or ANP, bone health and specialist falls assessment) being achieved consistently, a recommendation in the last national report^15^ indicated “The National Office of Clinical Audit (NOCA) should explore whether combining Irish Hip Fracture Standards (IHFS) 4–6 would appropriately measure best practice orthogeriatric care”. This work will be undertaken by the IHFD orthogeriatric network, who will also explore the need for future standards around the topics of nutrition and delirium screening as there continues to be a lot of variation in care described in those areas^15^.

## Discussion

Over the past decade, hip fracture care in Ireland has improved markedly. Although patient demographics have remained stable, clinical complexity has increased, yet performance against national standards has continued to rise. This progress reflects the combined impact of the IHFD, the BPT, and investment in orthogeriatric and multidisciplinary services, leading to better outcomes, more efficient pathways, and stronger organisational structures. This paper provides a comprehensive description of the governance, development, implementation, evolution and sustained momentum of the national hip fracture clinical audit between 2013 and 2024. It highlights the essential governance elements underpinning its success, including audit design and content, technical description, data entry and quality assurance, implementation and reporting/dissemination systems, funding, management, mandatory participation and data accessibility (Appendix 1).

The evolution of the IHFD has been characterised by a dataset that has remained relevant, responsive and informative. Audit outputs were co-designed with PPI contributors and stakeholders, ensuring meaningful translation into clinical practice and relevance. The high level of engagement, both at the operational level from NOCA and at hospital level from healthcare teams, were essential to sustaining. data attainment and continued participation.

The development of new services as the IHFD progressed is a clear indicator that the audit consistently focused on the right priorities. The introduction of the BPT drove consultant orthogeriatric and advanced nurse practitioner role developments. This change from minimal geriatrician involvement in hip fracture care in 2016 to orthogeriatric input in the majority of hospitals by 2020, highlights the influence of targeted incentives. Evidence internationally shows that clinical audit and feedback can drive measurable change and deliver better outcomes for patients [3], a trend reflected in this paper. Evaluation of the BPT in the UK between 2010 and 2016 demonstrated its impact, with 7600 lives saved and reductions in hospital admissions [33].

The Irish Hip Fracture Standards are the scaffolding from which the audit continues to drive improvements in patient outcomes. Time to surgery remains one of the most described targets in the literature [34] yet one of the most challenging to achieve in Ireland, despite national improvements in direct admissions through the national bypass protocol. Ireland’s compliance with time to surgery lags behind Sweden, Norway, UK, Australia and New Zealand [6]. An Irish study by Walsh et al. (2023) suggests that hip fracture patients with more comorbidity experience longer surgical delays and limited availability of senior orthopaedic surgeons contribute to surgical delays [35]. Variations in patient pathways, including via inter-hospital transfer or hospital bypass, require further exploration of their impact on achieving this standard.

Co-management of hip fracture patients by orthogeriatric teams has a positive effect on patient outcomes [36–38]. This is reflected in the IHFD data, which shows that despite increasing patient complexity more patients are being directly discharged home, achieving independent mobility prior to discharge and fewer require long-term care. The Irish Department of Health *Trauma System for Ireland* report, recognises orthogeriatrics as a key component of a national trauma system [39]. Data from the IHFD is shared routinely with the National Trauma Office, HSE to inform the reconfiguration of trauma services. The surge of improvements in bone health and specialist falls assessment are closely linked to the development of the orthogeriatric and ANP services. Secondary fracture prevention in this population is essential; between 1 and 9% of patients experience a contralateral hip fracture within one year and up to 20% over their lifetime [40, 41]. This risk coupled with Ireland’s ageing population begets the essential requirement to have a consistent model of orthogeriatric care in all hip fracture and trauma units.

Early mobilisation is one of the most modifiable factors associated with improving outcomes in hip fracture patients [42] and is associated with higher rates of direct discharge home [43]. This evidence informed the introduction of the seventh IHFS in 2018. Since its introduction, compliance with this standard has shown the greatest improvement of all IHFS standards and aligns with the expansion in seven-day physiotherapy services between 2016 and 2020. One of the most notable results has been the 50% reduction in patients requiring new admission into long-term care.

There is a clear focus within the IHFD on areas where high variation persists, including cognitive screening, nutrition, pain management, surgical site infections and longer-term outcomes at 30 days, 120 days, and 365 days post fracture [19]. To better understand variation in practice and resources across hospitals, an organisational survey was introduced in 2016. This survey served as a benchmark from which subsequent service and resource developments could be assessed.

Although 17 national hip fracture audits exist worldwide, there remains little published detail about their establishment, particularly in terms of funding, governance, management, leadership, QI frameworks, and the use of financial incentives such as the BPT, beyond the NHFD experience in the UK. This paper describes the practical complexity and addresses this gap by outlining the processes underlying the creation, evolution and continued momentum of the IHFD. It is expected that this detail will benefit established, new and emergent hip fracture audits internationally. The next phase of this work, will involve conducting a realist evaluation of the IHFD to identify the key mechanisms that enabled or constrained its progress. Realist methodology is increasingly used for exploring complex interventions’ successes and failures. The theory-driven approach seeks to explain “what works, how, why, in which contexts, for whom, and to what extent” through context–mechanism–outcome (CMO) configurations [44].

## Strengths and limitations

This paper describes a comprehensive account of establishing a national clinical audit using longitudinal data from 12 years, three organisational audits and expert knowledge from the author LB. No inferential causal analysis was undertaken. Rates and percentages reported are not adjusted for patient characteristics.

## Conclusion

This paper describes the establishment, implementation, evolution and sustained momentum of Ireland’s national hip fracture audit, detailing the funding model, governance structures, leadership approach, standards, analysis, reporting mechanisms and the implementation of a BPT. Over the past decade, the IHFD has driven improvements in data coverage and quality, compliance with care standards, patient outcomes and service development including informing national trauma service reconfiguration. We describe how a clinically-led, well-governed audit can deliver sustained improvement across the hip fracture care pathway. The transferability of findings from this paper to other audits in Ireland and globally will be enhanced by a subsequent realist evaluation of the IHFD’s development.

Continued investment in orthogeriatrics, advanced practice roles, and seven-day rehabilitation is essential to sustain progress. Protecting trauma capacity and improving timely access to surgery should remain priorities. Closer integration of IHFD data with national trauma planning and secondary fracture-prevention strategies will further strengthen a coordinated, system-wide approach to hip fracture care.

## Supplementary Information

Below is the link to the electronic supplementary material.

ESM 1 (DOCX 689 KB)

## Data Availability

All of the reports containing the data, organisational surveys and audit resources used in this paper are available at www.noca.ie.
