# Supplementary material for: The development and evolution of the Irish Hip Fracture Database: a quality care initiative 2013–2024
Source: Arch Osteoporos. 2026 Jun 25;21(1):96. doi: 10.1007/s11657-026-01731-8 (PMC13294304; doi:10.1007/s11657-026-01731-8)
Supplement: Supplementary file 1 — (DOCX 689 KB) [file 11657_2026_1731_MOESM1_ESM.docx]

**Appendix 1: Components and definitions of governance for Hip Fracture National Clinical Audit**

| **Component** | **Definition** |
| --- | --- |
| Construction of audit | How the audit was constructed (i.e., from clinical societies, through the health system, academic) |
| Content of audit | What was collected by the audit (i.e., patient demographics, standards, outcomes) |
| Technical description | What was the structure of the audit from an IT perspective (i.e., web- based, IT platform) |
| Data entry process | Who by and how was the data collected, i.e., clinical or admin staff, or directly from an electronic care record |
| Implementation process | How was the audit rolled out (i.e., pilot) |
| Data quality | Are there measures for assuring data quality i.e., data targets or standards reported |
| Reporting/data dissemination | How was the data shared back i.e., Annual reports, hospital reports, dashboards |
| Funding | How was the audit funded |
| Management | How is the audit managed and by whom |
| Mandatory participation | Is the participation in the audit mandatory or voluntary |
| Accessibility of data | How accessible is the data (i.e., research) |

**Appendix 2 Guidance for hip fracture governance committee**


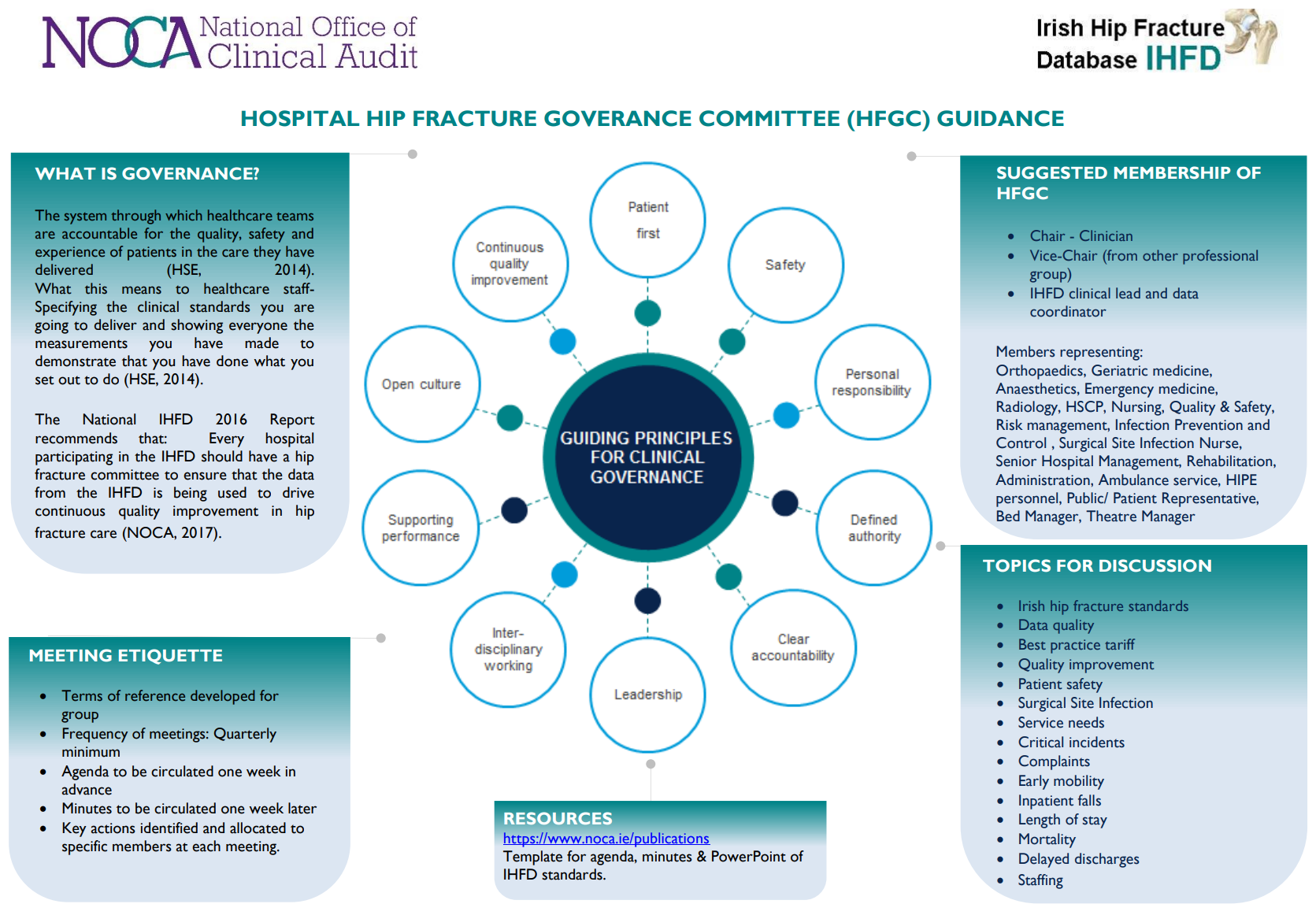


**Appendix 3: IHFD Data 2013-2024**
